# Accuracy of Machine Learning Models in Predicting Clinical Outcomes in Bipolar Disorder: A Systematic Review

**DOI:** 10.3390/brainsci16040415

**Published:** 2026-04-15

**Authors:** Jing Ling Tay, Ling Zhang, Kang Sim

**Affiliations:** 1 West Region, Institute of Mental Health, Buangkok Green Medical Park, 10 Buangkok View, Singapore 539747, Singapore; tay.jing.ling@aic.sg; 2 Singapore Institute of Technology, 1 Punggol Coast Road, Singapore 828608, Singapore; 3 Institute of Mental Health, Buangkok Green Medical Park, 10 Buangkok View, Singapore 539747, Singapore; ling.zhang@nhghealth.com.sg; 4 Yong Loo Lin School of Medicine, National University of Singapore, 10 Medical Drive, Singapore 117597, Singapore; 5 Lee Kong Chian School of Medicine, Nanyang Technological University, Clinical Sciences, Building, 11 Mandalay Road, Singapore 308232, Singapore

**Keywords:** artificial intelligence, treatment resistance, relapse, hospitalisation, remission, bipolar

## Abstract

Background/Objectives: Bipolar disorder (BD) is one of the leading causes of disability worldwide, causing significant functional impairments in those affected. The heterogeneous course of BD renders the prediction of clinical progress and outcomes challenging, but it can be potentially enhanced with the use of artificial intelligence methods. In this systematic review, we aimed to examine the extant literature regarding the predictive accuracy of clinical functioning, illness affective state, relapse, and relevant predictors amongst patients with BD, using artificial intelligence methods. Methods: The study was guided by PRISMA and the *Cochrane Handbook for Systematic Reviews*. Six electronic databases were systematically searched from inception for relevant studies until July 2025 and relevant data were summarised in tables. The protocol of the review was registered on Prospero, ID: CRD42024590343. Results: Forty articles were included in this review. The area under the curve (AUC) values for clinical functioning, illness affective state, and relapse prediction were 0.59–0.72 (poor to acceptable), 0.57–0.97 (poor to outstanding), and 0.45–0.98 (poor to outstanding), respectively. Supervised, tree-based algorithms performed the best. Predictive factors included sociodemographic, clinical and psychological factors and wearable data, as well as speech and video recordings. Conclusions: Existing studies showed the potential of machine learning methods in the prediction of clinical progress and outcomes of BD (specifically functional status, affective state, and relapse) based on relevant collected variables. Longitudinal studies can further clarify and validate the associated predictive factors for earlier identification of those at risk of poorer prognosis to enhance management of BD.

## 1. Introduction

Worldwide, bipolar disorder (BD) affects 37 million people [1,2]. BD is a major contributor to global disability and during manic episodes; patients with BD can experience irritability, increased energy, decreased need of sleep, impulsivity, racing thoughts, and other symptoms. During depressive episodes, they can experience suicidality, depressed mood, loss of interest, guilt feelings, lack of energy and concentration, and changes in sleep and eating patterns. A substantial proportion of patients with BD experience disruptions in their psychological and social functioning as a result of their condition [3]. Furthermore, BD is associated with increased mortality as compared with the healthy population due to premature death from suicide and other comorbidities such as cardiovascular, cerebrovascular, and respiratory conditions [4].

BD is heterogeneous in its types, presentations, course, and response to different treatments, thus rendering the prediction of clinical progress and outcomes challenging. In this regard, the use of artificial intelligence methods such as machine learning algorithms have been used to evaluate specific variables, which allow for better prediction of the clinical progress and outcomes of BD. For example, studies had sought to predict clinical functioning and changes in clinical states and illness course using sociodemographic, clinical, and other data [5,6,7]. Specifically, Rabeloo-da-Ponte et al. [8] examined 267 participants (167 outpatients with BD and 100 unaffected volunteers) from Brazil and Spain and classified the cognitive functioning of their cohort based on years of education, number of hospitalisations, and age. In another study conducted in Korea [9], sleep data (duration of sleep, length of circadian rhythm, step counts) from wearables were found to predict depressive, mania, and hypomania mood states. A study conducted in Spain by Anmella et al. [10] that evaluated data from wearables found that motor activities (acceleration), poor sleep, and skin temperature were most predictive of relapses. Miola et al. [11] found in their supervised machine learning model that cardiovascular, neurological, osteo-muscular disorders, valproate use, and age were associated with medical admissions in patients with BD.

To date, systematic reviews on machine learning applications in BD have been largely limited to diagnostic accuracy focused on diagnosis [12,13,14,15,16], while others have examined monitoring approaches for relapse [17,18]. Only one review to date has explicitly examined the prediction of relapse, hospitalisation, and suicide, but this was restricted to studies using neuroimaging and clinical data [19]. This review extends the existing literature by synthesising evidence across a broader range of clinically relevant outcomes, including clinical functioning and affective state, in addition to relapse, and by examining a wider spectrum of predictive variables, including clinical, psychological, behavioural, and physiological markers. Hence, we conducted a systematic review to evaluate the predictive accuracy of machine learning models in predicting clinical functioning, illness affective state, and relapse, as well as to identify the key predictors associated with these outcomes in bipolar disorder.

## 2. Methods

The methodology of this review paper was guided by the Preferred Reporting Items for Systematic reviews and Meta-Analyses (PRISMA) [20] and the *Cochrane Handbook for Systematic Reviews of Diagnostic Test Accuracy* [21]. The protocol of the review was registered on Prospero, ID: CRD42024590343. Our review questions were as follows:

1. What is the predictive accuracy for predicting clinical functioning, illness state, and relapse amongst patients with BD using machine learning methods?
2. What are the relevant clinical variables identified in predicting clinical functioning, illness state, and relapse among patients with BD?

Predictive accuracy was determined by measures including area under the curve (AUC), accuracy, and balanced accuracy, wherever available. The initial search strategy was conducted using PubMed with the key words ‘(artificial intelligence)’ AND (‘bipolar’) AND (‘functioning’ OR ‘illness state’ OR ‘relapse’). This initial search identified all relevant keywords specific to the PICO model. The PICO model is a framework used to formulate focused research questions by defining the Population, Intervention, Comparison, and Outcome. The following keywords were used:

(1) The keyword for population was ‘bipolar disorder*’.

(2) The keywords for machine learning methodology were (Artificial intelligence) OR (machine learning) OR (natural language processing) OR (neural network) OR (data science) OR (deep learning) OR (digital biomarker*) OR (digital phenotyping).

(3) The outcomes were (a) function*, (b) hospitali* OR relapse, OR (c) (illness state) (Supplementary Table S1). Keywords were searched on six electronic databases, Scopus, ScienceDirect, PsycINFO, PubMed, Embase, and CINAHL, from database inception until July 2025.

### 2.1. Study Selection

The study selection process followed a rigorous, multi-stage approach. Two authors independently assessed the identified articles from the database search. After the removal of duplicate records, the remaining articles were screened based on titles and abstracts to ensure a broad initial capture. Then, full-text screening was done for all potentially relevant articles to confirm eligibility based on methodology and clinical focus. Studies were included if they (1) applied machine learning methods for predictive modelling; (2) focussed on patients with bipolar disorder; and (3) predicted clinically relevant outcomes such as functioning, illness affective states, and relapse. Only peer-reviewed articles printed in English were included. Studies were excluded if they (1) did not employ machine learning techniques; (2) did not focus on bipolar disorder or include outcomes of interest related to bipolar disorder; (3) were review articles, editorials, commentaries, or conference abstracts without full methodological descriptions; or (4) did not provide sufficient methodological information for evaluation. In addition, reference lists of included articles were also reviewed to identify any additional studies not captured in the initial database search.

### 2.2. Data Collection and Analysis

The data summarised were clinical setting factors, participants’ characteristics, specific clinical outcomes examined (functioning, illness affective state, relapse), variables examined, and main findings (see Table 1). Predictive accuracy summaries are found in Table 2.

#### Risk of Bias

Risk of bias was appraised using the Joanna Briggs Institute checklist for diagnostic test accuracy studies [22]. The Joanna Briggs Institute checklist for diagnostic test accuracy studies was selected because included studies of prediction modelling evaluated the performance of machine learning models in predicting clinical outcomes, which involved the assessment of model performance measures such as accuracy and related metrics. The other framework such as the Prediction model Risk of Bias Assessment Tool (PROBAST) was also considered when interpreting the findings. Two authors appraised the risk of bias of included studies. Discrepancies between raters were resolved through mutual discussion within the study team to reach consensus.

## 3. Results

**Figure 1:**
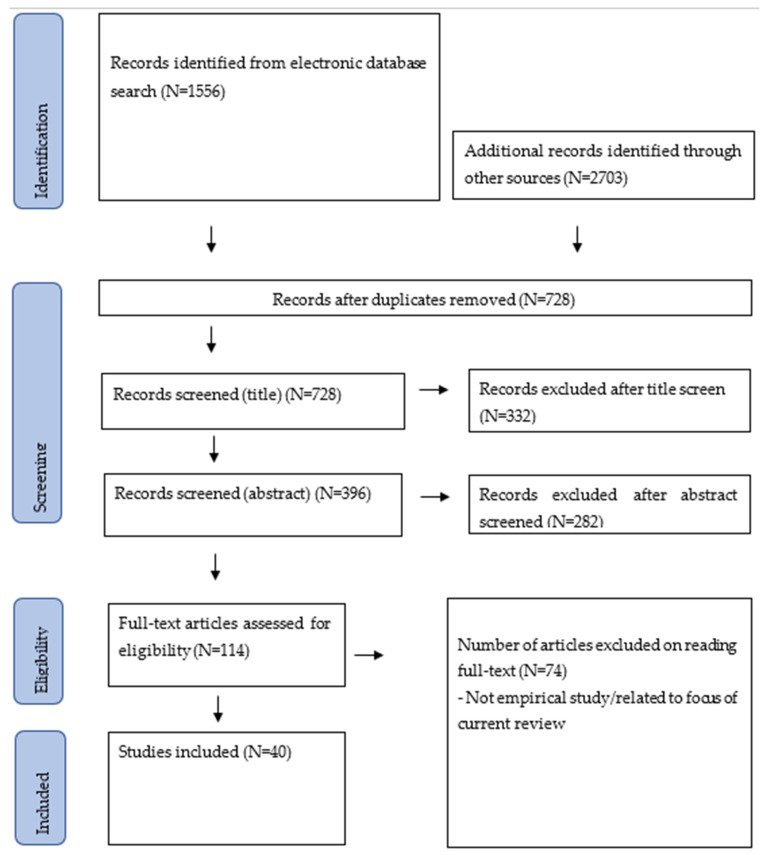
PRISMA chart.

Overall, a total of 1556 articles were initially identified, 114 full-text articles were evaluated, and 40 relevant studies were eventually included in this review (see Figure 1).

Regarding quality appraisal of the included articles, the Joanna Briggs Institute checklist for diagnostic test accuracy studies was used and three items (blinded interpretation of both the index test and reference standard results; appropriate interval between index test and reference standard) were excluded because they fall outside the scope of this review, focusing on clinical diagnostic accuracy rather than internal validation of artificial intelligence methods. Scores of zero to three are considered poor quality, while scores of four and above indicate moderate quality. The scores of the quality appraisal for the included papers (see Supplementary Table S2) ranged from three to seven, with most studies scoring about four to five (moderate quality).

The main details of the included studies are summarised in Table 1. In terms of the sites of the studies, the majority of studies (n = 26, 65%) were conducted in the West, namely (1) Americas (11 studies) such as the United States (n = 7) [23,24,25,26,27,28,29], Colombia (n = 1) [30], and Brazil (n = 3) [5,31,32], and (2) Europe (15 studies) such as Austria (n = 3) [33,34,35], Greece (n = 2) [36,37], Italy (n = 2) [11,38], Norway (n = 2) [39,40], Spain (n = 2) [10,41], Denmark (n = 1) [42], and Portugal (n = 1) [43]. One study recruited from more than one country, i.e., Brazil and Spain [8], and another study was based on a European project [44].

The rest of the included studies were from Asia (14 studies) such as Korea (n = 2) [9,45], China (n = 2) [7,46], Japan (n = 1) [47], and India (n = 1) [48]. Three studies recruited participants from a Turkish database [49,50,51], and three studies from the Audio Visual Emotion Challenge dataset [52,53,54]. One study recruited participants in Jordan (n = 1) (Ebrahim et al., 2018) and another from those who accessed a phone application (BiAffect) [6].

In terms of sample size, it ranged from six to 20,000 participants across the studies. The number of electronic health records and call entries ranged from 3085 to 77,296. Predictive items that were examined included sociodemographic and clinical variables (psychiatric, medical history factors) and data from pharmacological, biochemistry, MRI, social, psychological, physiological (sleep, activity), speech (acoustic, linguistic), and video (facial cues, eye gaze) domains.

**Table 1:** Details of included studies.

	Authors/Year/Country/Setting	Variables Measured	Main Findings
	**Functioning**		
1	(de Aguiar et al., 2023) [31] Brazil Population-based cohort study	Sociodemographic, behavioural, and environmental data to predict functional impairment (n = 282).	Variables predictive of poor functioning were sexual abuse during childhood, severe anxiety, depressive and somatic symptoms, emotional and physical abuse, and physical neglect during childhood.
2	[32] Brazil Outpatient clinic of a general hospital	Sociodemographic, clinical, MRI, and functioning (Functioning Assessment Short Test) data (n = 35).	Functioning was predicted by volumes of specific brain structures including the left superior frontal cortex, right white matter, left rostral medial frontal cortex, and right lateral ventricle volume.
3	[8] Brazil, Spain Outpatient clinics	Clinical and sociodemographic data (questionnaire), and neuropsychological battery by psychologists (n = 267 participants).	Three cognitive subgroups: (1) Intact (35%) (more education, lower hospitalisation); (2) Selectively impaired (35%) (younger age, fewer education); (3) Severely impaired (30%) (older age, fewer education). Variables that could discriminate the subgroups were years of education, numbers of hospitalisation, and age. Other variables not discriminative were age of onset, years of disease, family history of mood disorders, and first-episode psychosis.
	**Illness affective state differentiation**		
4	[5] Brazil Institute of Psychiatry of the University of São Paulo Medical School outpatient clinic	Demographic and clinical variables (n = 148, 75 predominantly depressive, 73 predominantly maniac).	Random forest selected 13 important features, although only 5 showed significant group differences. The significant variables were the number of manic, hypomanic, depressive episodes, past hospitalisation, and history of hospitalisation in differentiating manic predominant polarity or depressive predominant polarity. The non-significant variables were the number of total or mixed episodes and history of suicide attempts. Presence of anxiety disorders, substance dependence, and eating disorder were also non-significant.
5	[6] Jordan Secondary analysis of Bipolar Disorder Challenge	Visual–audio recordings.	Accuracy values of test and development were negatively correlated when differentiating remission, hypomania, and mania. Poor performance for external validation.
6	[7] China Hospital	Electronic health records (n= 3085).	Mania patients had higher blood pressure, pulse, and temperature. Depressed patients had more comorbidities, anxiety, and sleep problems. Patients with mixed state had worse suicidal thoughts and psychometric impairments.
7	[23] United States Longitudinal dataset from University of Michigan	Speech recordings from phone conversations (n = 37, 34,830 calls from 2436 h).	Processes such as declipping and segmentation are essential to minimise the varying effects of loudness, noise, and clipping to provide the best results. Samsung S5 outperformed Samsung S3 in predicting mania.
8	[24] United States Database of longitudinally collected audio data	Speech recordings (n = 221.2 h of 3588 phone calls).	Vocalisation and pause ratio measure was reduced in depressed speech and greater in hypomanic speech.
9	[33] Austria Psychiatric hospital	Subjective mood state, psychological tests, as well as objective sensor data from smartphone (n = 10).	In differentiating depressive, euthymic, and manic states, pure phone call data performed worse than phone call and voice data. Additionally, acceleration and GPS data provides better accuracy in prediction as compared to sound and phone call data.
10	[34] Austria Psychiatric hospital	Audio and motor (speed of picking phone up, initiating and ending the call) activities (n = 10 participants).	In differentiating depressive, maniac, and euthymia states, the best results included emotional and spectral audio features and frequency captured from accelerometers. In depressive state, there was reduced speaking rate, as well as increased respond delays and average pauses.
11	[35] Austria Psychiatric hospital	Speech data from phone calls (includes emotional acoustics) (n = 6 participants).	Most important features were length of phone conversations, average harmonics-to-noise ratio, average utterances, pitch (stress detection), and daily phone call length in differentiating depressive or manic episodes.
12	[38] Italy Outpatient and inpatient services	Speech (acoustic, linguistic features) (n = 37).	Speech data (duration and number of pauses) were able to classify mood state better than speech duration/rate. Notable gender differences in mood expression via linguistic and acoustic features.
13	[39] Norway Haukeland University Hospital	Motor activity from wearable (n = 16 participants).	Motor activity was obviously deviated as compared with periods of euthymia. Unstable activity patterns noted in mania, and dramatic activity shifts noted in euthymia.
14	[44] Data from a European Project	T-shirt wearable that captures physiological data, ECG, and body posture (n = 8 participants).	In differentiating remission, depressive, hypomania, and mixed states, continuous and personalised monitoring achieved an accuracy of up to 95.81%.
15	[45] Korea Korea University Hospital	Demographic, clinical, subjective mood state, and smartphone data (n = 55).	Activity and light exposure were greater in the high-mood group at night and in the low-mood group in the daytime. High-mood group also had sleep irregularity. Total sleep time and sleep quality did not distinguish between both groups.
16	[48] India	Speech (acoustic and linguistic cues) and visual (eye gaze, facial head posture, facial landmarks) data (n = 164 participants).	In differentiating remission, hypomania, and mania, integration of variables of multiple modalities (verbal, visual, aural) improved accuracy. Fusion models performed well even without visual variables.
17	[49] Turkish database	Facial cues from video recordings (n = 47, 218 recordings).	The CNN with Long Short-Term Memory classifier model outperforms previous examined algorithms, including other visual features in differentiating remission, hypomania, and mania.
18	[50] Turkish database	Speech recordings (n = 46, 222 recording).	The novel stacked ensemble classifier model outperforms previous examined algorithms for the test set in differentiating remission, hypomania, and mania.
19	[51] Turkish database	Speech recordings (n = 46).	AUDEEP, a type of Recurrent Neural Network, was the best performing algorithm. CapsNets (Capsule Neural Network) produced promising results without hyperparameter optimisation in differentiating remission, hypomania, and mania.
20	[52] Audio Visual Emotion Challenge (AVEC2018) dataset	Audio and visual features (n = 46 participants).	Body posture and head movements are important features that improved the accuracy value in differentiating remission, hypomania, and mania. Captures of minor movements of facial landmarks were more useful than capturing large range motions.
21	[53] Audio Visual Emotion Challenge (AVEC2018) dataset	Audio and visual features (n = 47 participants).	A new hierarchical model using audio (tone, rhythm, pitch) and visual features (eye movements) that interprets varying levels of mania outperformed previous methods.
22	[54] Audio Visual Emotion Challenge (AVEC2018) dataset	Audio and visual features.	Audio features perform better than video features. Emotions in speech data were vital and could classify bipolar state better in males than females, implying that manic features in males may be more overt. Upper body posture examined with Histogram of Displacement achieved effective results in differentiating remission, hypomania, and mania.
23	[55] BiAffect application users	Passive technology interactions such as keystroke metadata (n = 328) to detect variations in PHQ.	Higher rates of typing errors and keypresses predict depression. Young age, female, higher likelihood of post-traumatic stress disorder, and attention deficit hyperactivity disorder were predictive of PHQ score variations.
	**Illness Relapse**		
24	[9] Korea 8 hospitals	Subjective mood and energy data, and physiological and circadian rhythm data from Fitbit wearable and smartphones.	Digital phenotypes depicting circadian disruption was most indicative for depression and hypomania prediction, and step counts for mania. Predictive features were: - Depression (reduced sleep efficacy, greater standard deviation of length of sleep, greater mean steps at night, lower mean steps in daytime, greater circadian rhythm acrophase). - Mania (greater step count at night, shorter sleep, greater sleep length standard deviation, greater circadian rhythm acrophase). - Hypomania (greater step count at night, shorter sleep, less morning light exposure, shorter mean circadian rhythm acrophase).
25	[25] United States Academic hospital	Sociodemographic and clinical data and Borderline Personality Questionnaire (n = 71).	Young age, female gender, reduced length of stay, higher scores on the Borderline Personality Questionnaire Self-Image and Suicide/Self-mutilation subscales, and higher prevalence of prior hospitalisations were associated with greater risk of hospitalisations.
26	[26] United States	Clinical, demographic and MRI data (n = 81).	Severity of depressed mood was predicted by connectivity of supplementary motor area nodes and left dorsolateral prefrontal cortex with various cortical, motor, limbic, and cerebellar areas. Severity of elevated mood was predicted by connectivity of right visual association region nodes and left fusiform with various insular, motor, limbic, and posterior cortices.
27	[36] Greece University Mental Health	Physiological data (activity, movement, heart rate) from wearables (n = 24 patients with psychotic or affective disorders).	Patients with mild relapses yielded worse detection performance than moderate and severe relapses, while reconstruction error was higher during moderate and severe relapse periods.
28	[37] Greece Research Institute	Wearable that recorded biometric and behavioural factors (n = 24 patients with psychotic or bipolar disorder; n = 23 healthy controls).	Patient group had greater variability for movements (acceleratory, gyrometer). Patients showed also had altered heart rate variability patterns—with less complex heart rate dynamics and a different balance between frequency bands—compared to healthy people.
29	[10] Spain Inpatient, patients’ homes, or outpatient settings	Physiological data from wearable recordings across 3 episodes (acute, responding to treatment, remission); patients (n = 12); controls (n = 7); 1512 h of data.	Electrodermal activity (aggressiveness, anxiety), acceleration (motor activities, poor sleep), and skin temperature were most predictive.
30	[42] Denmark Data from Bipolar Illness Onset study	fMRI when conducting an emotional regulation activity. Patients with bipolar disorder (n = 87); controls (n = 66).	Increased amygdala activity had greater relapse risk. 1st group: Elevated bilateral amygdala reactivity and normal temporo-parietal and prefrontal activation. 2nd group: Hypoactivity in all emotional regulation areas. Patients had longer and more frequent mixed affective episodes as compared to the 1st group.
	**Depressive relapses**		
31	[27] United States 20 sites	Secondary analysis of national clinical data (n = 800, 507 had relapse).	Anhedonia, days of dysphoria, and fatigue were most important features. Other features included low self-esteem, psychomotor retardation, concentration, sleeping problems, guilt, and poor appetite. Less vital features were risky behaviours and inability to participate in new projects.
32	[43] Portugal University outpatient clinic	109 variables including mood symptoms and medications (n = 108, 86 had relapse).	Young age of onset and greater years of diagnosis. Non-significant variables: Gender, age, years of education, race, and type of bipolar (I versus II). Psychiatric comorbidity, psychotic symptoms, suicidal attempt, and rapid cycling status.
33	[47] Japan 10 inpatient and outpatient psychiatric units	Videos of upper body motion using sensors, n = 47 (bipolar 14, depression 17, healthy control 16).	Slower head movements were noted amongst patients with depression.
	**Manic relapse**		
34	[40] Norway Affective inpatient and outpatient units	Physiological data from wearable, including acceleration; n = 47 (25 euthymia, 22 mania).	Hand gesture recognition, actigraphy, and heart rate are important features.
35	[41] Spain 4 hospitals	Clinical and fMRI data.	Schizoaffective disorder diagnosis, younger onset of age, symptoms such as impulsivity, abnormal thought process, and reduction in inferior frontal white matter or cerebellum volume. Estimations with both MRI and clinical data improved estimation.
36	[46] China Hospital	Speech data (n = 21 participants).	Mania in single and multiple patients were best detected by Support Vector Machines and Gaussian Mixture Model, respectively.
	**Hospitalisation**		
37	[28] United States University of California Health Care System	Demographic, clinical, and pharmacological data from electronic health records (n = 77,296 notes).	(a) Length of stay > 5 days, medical diagnosis at first encounter, multi-comorbid disorders, and discharge to places besides facilities. (b) Caucasian, length of stay < 6 days, with >12 E room visits last year. (c) Male, transferred between medical and psychiatric services, and length of stay < 3 days.
38	[29] USA VA Health system	Clinical data including diagnoses, laboratory results, parameters, and pharmacological treatment (n = 20,000 patients).	The prediction of mortality was better than hospitalisation. The prediction of hospitalisation related to medical reasons was better than hospitalisation prediction due to mental health reasons. Hospitalisation due to alcohol-related reasons had the best predictive accuracy.
39	[30] Colombia Outpatients from Sanitas healthcare facilities	Sociodemographic, clinical, and medical data from electronic health records (n = 2726 patients).	Younger patients, symptoms of borderline personality disorder, prior E room visits, medical history of hypertension, chronic obstructive pulmonary disorder, and hypothyroidism.
40	[11] Italy Mental health unit of Padova University Hospital	Sociodemographic, clinical, laboratory, and medication data and medical history (n = 71 participants).	The top performing model that utilised 109 sociodemographic, clinical, ECG, and laboratory data found that the top predictive variables for medical admissions were cardiovascular, neurological, and osteo-muscular disorders, as well as valproate and age. The second model that utilised medical variables found that top variables were cardiovascular and neurological disorders, as well as deep vein thrombosis, osteo-muscular disorders, infectious disease, and age. A third model that used sociodemographic and psychiatric variables performed poorly.

CNN = convolutional neural network; ECG = electrocardiogram; fMRI = functional magnetic resonance imaging; MLP = Multi-Layer Perceptron, NAS = Neural Architecture Search; PHQ = Patient Health Questionnaire; sig = significant; bold and underline = outcome variables.

### 3.1. Accuracy of Artificial Intelligence Methods in Predicting Clinical State or Outcomes

Predictive accuracies of studies are reported with accuracy, balanced accuracy, and/or area under the curve (AUC) (Table 2). AUC classifies true negatives and positives [56] with the following scorings: poor (0.50 to <0.70), acceptable (0.70 to <0.80), excellent (0.80 to <0.90), and outstanding (0.90 and above) [57]. Predictive accuracies are more specific for classification models in machine learning and include accuracy and balanced accuracy. Accuracy is the number of correct predictions divided by the number of total prediction number. Balanced accuracy is reported for imbalanced data and is the division of (sensitivity + specificity) by 2. The interpretation of scorings for accuracies are poor (<60%), acceptable (60% to <70%), good (70% to 90%), and very good (>90%) [58].

The included studies showed substantial heterogeneity in data sources, machine learning algorithms, outcome definitions, and predictors. Similarly, a wide variety of supervised algorithms was applied, and outcomes such as clinical functioning, affective state, and relapse were defined and operationalised differently across studies. As a result, while comparative summaries of predictive performance provide a useful overview of the field, differences across studies should be considered when interpreting variations in reported metrics.

Illness affective state prediction had the highest predictive accuracy, achieving an AUC of 0.57–0.97 (poor to outstanding) and accuracy of 45–100% (poor to very good), while illness relapse prediction had an AUC of 0.45–0.98 (poor to outstanding) and accuracy of 52–98% (poor to very good). Predictive accuracy for clinical functioning was the lowest, achieving an AUC of 0.59–0.72 (poor to acceptable) [31,32]. The AUC values in this review showed greater variability compared to those reported in another review examining relapse (AUC 0.71–0.98) and suicide (AUC 0.71–0.99) in bipolar disorder [19]. This likely reflects the greater heterogeneity of the included studies in the present review, whereas Amanollahi et al. [19] primarily included studies based on neuroimaging and clinical data that largely utilised internal validation. In addition, the present review includes a broader range of outcomes, such as clinical functioning and affective state, and captures emerging multimodal approaches, including wearable, behavioural, and speech data.

**Table 2:** Machine learning algorithms/models used and associated predictive accuracies of specific clinical outcomes.

Studies	Outcome	Predictive Variables	Algorithms/Models	AUC	Accuracy/Balanced Accuracy	Other Measure ^2^ = RMSE; ^3^ = Unweighted Average Recall
	**Functioning**					
[31]	Functional impairment	Sociodemographic, behavioural, and environmental data	**Random forest**	**0.68–0.72**		
			LASSO	0.59–0.69		
[32]	Functioning	Sociodemographic, clinical, and MRI data	Support Vector Regression			15.09 ^2^
	**State**					
[5]	State	Demographic and clinical data	Random forest	0.75	65%	
[6]	State	Visual–audio recordings	Mel frequency cepstral coefficient			49% ^3^
			eGeMAPS			55% ^3^
			Bag-of-Audio-Words			55% ^3^
			DeepSpectrum			58% ^3^
			Facial action units			56% ^3^
			Bag-of-Video-Word			56% ^3^
			eGeMAPS + Facial action units			60% ^3^
			DeepSpectrum + Facial action units			63% ^3^
			Bag of Words (Concatenated)			64% ^3^
			All (Concatenated)			59% ^3^
			**Fusion (Mel frequency cepstral coefficient + Bag-of-Video-Word)**			**75% ^3^**
[7]	State	Electronic health records	Support Vector Machine	0.74	75%	
			**Extreme Gradient Boosting**	**0.80**	**80**%	
			Logistic regression	0.76	77%	
			Random Forest	0.72	78%	
			Resampling of train data:			
			Support Vector Machine	0.74	74%	
			**Extreme Gradient Boosting**	**0.79**	**74**%	
			Logistic regression	0.73	63%	
			Random Forest	0.76	75%	
[23]	Mania state	Speech recordings	Support Vector Machine	* 0.57–0.74		
	Depressed state		**Support Vector Machine**	* 0.64–0.77		
[33]	State	Subjective mood state and psychological tests, as well as objective sensor data from smartphone	Naive Bayes (phone call features)		66%	
			**Naive Bayes (sound features)**		**70%**	
			Naive Bayes (fusion)		69%	
[34]	State	Audio data from call activities	C4.5		77%	
			Random Forest		70%	
			Support Vector Machine		70%	
			Naïve Bayes		48%	
			K Nearest Neighbour		69%	
			AdaBoost		49%	
			**Bagging**		**79%**	
		Spectral features	C4.5		77%	
			**Random Forest**		**80%**	
			Support Vector Machine		70%	
			Naïve Bayes		47%	
			K Nearest Neighbour		59%	
			AdaBoost		49%	
			Bagging		79%	
[35]	State	Speech data	^ Random Forest (phone call statistics)			77% ^1^
			^ Random Forest (speaking parameters)			78% ^1^
			^ Random Forest (emotional acoustics)			79% ^1^
			**^ Random Forest (all)**			**82% ^1^**
[38]	State	Speech data	**Neural network (natural language processing)**	**0.85**	71%	
			Neural network (acoustic: shimmer/jitter-related)	0.61	54%	
			Neural network (speech duration/rate)	0.70	65%	
			Combined	0.65	71%	
[8]	State	Clinical, sociodemographic, and neuropsychological data	Classification and Regression tree	0.61	62%	
[44]	State	Physiological data, ECG, and body posture	Support Vector Machine (standard)		56–81%	
			**Support Vector Machine (Markov)**		**78–96%**	
[45]	State (no episode)	Demographic, clinical, subjective mood state, and smartphone data	Random forest	0.87	85.3%	
[48]	State (mania, hypomania, remission)	Speech data	One-Class Support Vector Machine	0.75	48%	
			Kennel Extreme Learning Machine	0.78	53%	
			eGeMAPS10 (Acoustic)	0.82	67%	
			Linguistic Inquiry and Word Count	0.76	55%	
			Facial Action Units	0.77	60%	
			**Multimodal model**	**0.85**	67%	
[52]	State	Audio and visual data	Convolutional Neural Network: Motion History Histogram feature on facial landmarks		57%	
			Convolutional Neural Network: Histogram of Displacement range on facial landmark		46%	
			**Convolutional Neural Network: Histogram of Displacement range on body posture**		**66%**	
			Convolutional Neural Network: Motion History Histogram feature on body posture		52%	
			Convolutional Neural Network: Audio-functional		55%	
[53]	State	Audio and visual features	**Gradient Boosted Decision Tree (audio + video)**		**100%**	
	Remission, hypomania		Gradient Boosted Decision Tree (audio)		87%	
	Hypomania, mania		Gradient Boosted Decision Tree (audio)		98%	
	Remission, hypomania		**Gradient Boosted Decision Tree (video)**		**100%**	
	Hypomania, mania		Gradient Boosted Decision Tree (video)		76%	
[54]	State	Audio and visual features	**Decision Fusion**		**78%**	
			Model Fusion		72%	
[55]	State (variations in PHQ, depression)	Passive technology interactions such as keystroke metadata	**Random forest**	**0.97**	**90%**	
			Neural Network	0.93	87%	
			Gradient Boost	0.95	89%	
			Support Vector Machine	0.91	82%	
	**Relapses**					
[9]	Depressive relapse	Subjective mood and energy data and physiological and circadian rhythm data from wearable and smartphone	Random Forest	0.95–0.96	92%	
	Maniac relapse		Random Forest	0.97	94%	
	Hypomania relapse		**Random Forest**	**0.97–0.98**	**93–95%**	
[24]	Hypomania	Speech recordings	**SVM with linear and radial-basis-function**	**0.81**		
	Depression			0.67		
[25]	Rapid readmission	Sociodemographic and clinical data, and Borderline Personality Questionnaire	Support Vector Machine	0.86		
[26]	Depressive relapse	Clinical, demographic, and MRI data	Connectome-based Predictive Modelling (both positive and negative network)			3.5 ^2^
			Connectome-based Predictive Modelling (negative network)			3.3 ^2^
	Mania relapse		Connectome-based Predictive Modelling (both positive and negative network)			5.8 ^2^
			Connectome-based Predictive Modelling (negative network)			5.9 ^2^
[27]	Depressive relapse	National clinical data	Support Vector Machine			74% ^1^
			**Random Forest**			**80% ^1^**
			Naïve Bayes			65% ^1^
			Multi-Layer Perceptron			51% ^1^
			Logistic Regression			66% ^1^
[28]	Admission	Demographic, clinical, and pharmacological data	Classification and regression trees	0.87–0.88	88–98%	
[29]	Admission + mortality	Clinical data	**Deep Neural Network**	**0.75–0.81**	**77**–**95**%	
			Support Vector Machine	0.74–0.80	76–95%	
[30]	Admission	Sociodemographic, clinical and medical data	Decision tree	0.85–0.91	91–93%	
			**Random Forest**	**0.98**	**93–95%**	
			Logistic regression	0.94–0.96	91–93%	
			Support Vector Machine	0.97–0.98	92–95%	
	Readmission		Decision tree	0.52	55%	
			**Random Forest**	**0.58**	**55%**	
			Logistic regression	0.48	55%	
			Support Vector Machine	0.50	52%	
[36]	Relapse	Physiological data from wearables	Transformers	0.45		
			Fully connected Neural Networks	0.47		
			Convolution Neural Networks	0.51		
			**Gated Recurrent Unit**	**0.53**		
			Random (no training)	0.50		
[37]	Bipolar relapse	Wearable that recorded biometric and behavioural data	Fully connected Neural Network	0.47		
			Convolution Neural Network	0.51		
			Transformer	0.45		
			**Gated Recurrent Unit**	**0.53**		
			Random Forest	0.50		
[11]	Hospitalisation	Sociodemographic, clinical, laboratory, medication, and medical data	**Lasso regression (Global model)**	**0.70**		
			Lasso regression (Mental health model)	0.62		
			Lasso regression (Medical model)	0.69		
[40]	Manic relapse	Physiological data from wearable	Short Network	Manic relapse	88%	
			Short-Long Network		91%	
			Short Ensemble Networks		94%	
			**Short-Long** **Ensemble Networks**		**97%**	
[10]	Severity of relapse (depression)	Physiological data	**Bidirectional Long Short-Term Memory (RNN)**	0.61	62%	
	Severity of relapse (mania)			**0.70**	**70%**	
	Severity of relapse (mixed state)			0.63	63%	
[41]	Manic relapse	Clinical and fMRI data	**Cox LASSO model (MRI + clinical)**	**0.61–0.66**		
			Cox LASSO model (MRI only)	0.49–0.58		
			Cox LASSO model (clinical only)	0.51–0.55		
[43]	Depressive relapse	Mood symptoms and medication history	Inductive Logic Programming		85%	
[45]	Depressive relapse	Demographic, clinical, subjective mood state, and smartphone data	Random forest	0.87	87%	
	Manic relapse		**Random forest**	**0.96**	**94%**	
	Hypomanic relapse		Random forest	0.91	91.2%	
[46]	Mania relapse	Speech data	Support Vector Machine			
			**Gaussian Mixture Model**		**72%**	
[47]	Depression relapse	Videos of upper body motion using sensors	Support Vector Machine with radial basis function kernel		72–95%	
[49]	Non depressive relapse	Facial cues from video recordings	Inductive Logic Programming		91%	
[55]	Mania relapse	Passive technology interactions such as keystroke metadata	Actigraphy + Heart Rate Variability Long Interval		90%	

eGeMAPS = Extended Geneva Minimalistic Acoustic Parameter Set; PHQ = Patient Health Questionnaire; ^1^ = F measure, ^2^ = Root Mean Square Error (RMSE); ^3^ = Unweighted Average Recall; MLP and NAS; RNN = recurrent neural network; bold and underline = outcome variables; bold = best performing machine learning model; ^ random forest performed better than SVM and neuronal network; * Samsung S5 performed better than Samsung S3 phone.

Among the studies that evaluated the performance of several algorithms, random forest consistently outperformed the other algorithms in five studies [27,30,31,34,55], achieving an AUC of 0.68–0.98 (poor to outstanding) and accuracy of 80–95% (good to very good). Another tree-based algorithm, gradient boosting, also performed well in two studies, as reflected by an AUC of 0.79–0.80 [7] and accuracy of 80–100% [53].

### 3.2. Functioning

Three studies assessed functioning as an outcome [8,31,32]. Sociodemographic and clinical data that were predictive of better functioning included higher education levels, fewer hospitalizations, and specific brain structures such as larger frontal cortex volume and smaller right lateral ventricle volume [32] Poorer functioning was associated with childhood abuse and neglect [31].

### 3.3. Illness Affective State Differentiation

Twenty studies evaluated variables associated with illness affective state. Two studies examined demographic and clinical variables [5] while one study evaluated data from electronic health records [7]. One study examined video recordings [49] and five studies examined speech recordings [23,24,38,50,51], whereas another five studies examined both video and speech recordings [6,48,52,53,54]. Four studies examined data captured by smartphone, including physiological [33,45] and phone activity [34,35]. Two studies evaluated physiological data from wearables [39], including a T-shirt wearable [44], while another study examined the keystroke data from BiAffect application users [55].

#### Predictors of Mood State

(i) Sociodemographic and clinical data

Relevant associated clinical variables included a history of hospitalisation, number of hospitalisations, and number of hypomanic, manic, or depressive episodes [5]. Conversely, the number of total episodes or number of mixed episodes did not differentiate mood state, nor did the number of suicidal attempts or co-morbidities such as substance dependence, anxiety, and eating disorders [5].

(ii) Video and speech recordings

For speech recordings, one study found that pause features (duration and number of pauses) could classify mood state better than duration and rate of speech [38]. Likewise, reduced pause ratio measure and greater vocalisation were found in hypomanic speech, while the reverse was true for depressed speech [24].

Several studies evaluate variables that can distinguish between mania, hypomania, and remission. A study using video recordings found that body posture and head movements were important variables [52]. The study also observed that minor, rather than large, movements were useful variables [52]. Conversely, another study found that integrated variables of various modalities (verbal, visual, and aural) performed the best [48]. Yet another study found that speech data was more important than video data [54]. The study also found that emotions in speech data could classify states in males better than in females [54].

(iii) Smartphone and wearables

In terms of data from wearables, voice, together with phone call data, performed better than phone call data alone [33,35]. However, activity data performed better than voice and phone data [33]. Notably, another study found that activity, together with voice and speech data, performed the best [35].

Mania state was observed to have unstable patterns of activity while euthymic state was associated with dramatic activity shifts [39]. Activity and light exposure were observed to be higher at night in the high-mood group, but higher during the day in the low-mood group [45]. Sleep irregularity was also prevalent in the high-mood group [45]. Among a group of BiAffect application users, variables predictive of mood variations were female, younger age, post-traumatic stress disorder, and attention deficit stress disorder [55].

### 3.4. Relapse

Seventeen studies evaluated relapse as an outcome. Three studies specifically examined depressive [27,43,47] and manic relapses [40,41,46], while four studies examined hospitalisation [11,28,29,30].

Seven studies examined sociodemographic and clinical data [11,25,27,28,29,30,43], one study examined MRI data [42], and two studies examined both sociodemographic and clinical data, as well as MRI data [26,41]. One study each examined video recordings [47] and speech recordings [46]. Six studies examined information from wearables including physiological data [9,10,36,37,40] and subjective mood [9].

#### Predictors of Relapse

(i) Sociodemographic and clinical data

Sociodemographic characteristics predictive of relapse included younger age [25] and both female [25] and male gender [28], in contrast to another study which did not find associations with gender, age, ethnicity, and years of education [43].

For clinical data, younger age of onset [41,43], longer duration of illness [43], frequent hospitalisations or emergency room visits [25,28], short hospitalisation stays [25,28], multiple comorbidities [28], disorders of cardiovascular, neurological and osteo-muscular systems [11], greater symptoms of borderline personality disorder [25], administration of valproate medication [30], and discharge to non-facility settings [28] were relevant predictors of relapse.

In terms of symptomatology, anhedonia [27], fatigue [27], impulsivity [41], and abnormal thought process [41] were associated predictors compared with low self-esteem, psychomotor retardation, poor concentration, sleeping problems, and poor appetite [27].

In terms of brain MRI data, increased amygdala activity [42] and reduction in inferior frontal white matter or cerebellum volume [41] were associated predictors of relapse. Severity of depressed mood was predicted by connectivity of supplementary motor area nodes and left dorsolateral prefrontal cortex with various cortical, motor, limbic, and cerebellar areas while severity of elevated mood was predicted by connectivity of right visual association region nodes and left fusiform with various insular, motor, limbic, and posterior cortices [26]. Patients with longer and more frequent mixed affective episodes had hypoactivity in emotional regulation areas [42].

(ii) Video recordings

In terms of video recordings, slower head movements were noted in people experiencing depression [47].

(iii) Wearables

Associated predictors from wearables included electrodermal activity (aggressiveness, anxiety) [10], acceleration (poor sleep, motor activities) [10,37,40], heart rate [40], skin temperature [10], and hand gestures [40]. More specifically, in depression, there was reduced sleep efficacy with greater mean steps at night but lower mean steps during the day, with greater circadian rhythm [9]. In mania, there was shorter sleep with similarly greater mean steps at night and circadian rhythm acrophase. Hypomania presented similarly to mania, except that it showed a shorter mean circadian rhythm phase [9].

## 4. Discussion

This review found that most of the included studies were from the West (65%) and mainly published in the last six years. Several key findings were noted. First, the range of predictive accuracies using machine learning models was relatively wide for functioning, affective state, and illness relapse. Second, the best performing method was supervised model tree-based algorithms, particularly random forest and gradient boosting. Third, heterogeneous predictive variables examined included data related to sociodemographic and clinical factors, pharmacological and MRI data, physiological measures, and speech and video recordings.

Overall, AUC values for prediction of affective state, illness relapse, and functioning were 0.57–0.97 (poor to outstanding), 0.45–0.98 (poor to outstanding), and 0.59–0.72 (poor to acceptable) respectively. The values were comparable with other studies using machine learning methods, albeit to diagnose BD (AUC 0.48–0.65) and schizophrenia (AUC 0.54–0.95).

The wide range of predictive performance observed across studies appears to reflect heterogeneity in outcome definitions, data sources, and modelling approaches, rather than random variation. Higher predictive accuracy was more frequently reported in studies using digital phenotyping or electronic health record data, particularly for affective state, relapse, and hospitalisation outcomes that rely on behavioural variables, while prediction of functional outcomes and single-modality models showed comparatively more modest or variable performance. Affective state prediction yielded the narrower range of predictive accuracies compared with functioning, as varying mood states may be associated with relevant clinical symptoms and physiological measures. Of note, prediction of relapse relies on the relevant combination of baseline measures for clinical progress over time. Prediction of functioning as an outcome variable was limited by the small number of studies to date and may not capture significant data points such as support system, personal coping, and intercurrent stressors within the machine learning studies. Likewise, several studies reported very high AUC values (0.97–0.98). These findings should be interpreted with caution due to limitations such as small datasets, lack of external validation, and potential overfitting. Consequently, such results might not be generalisable to external datasets or clinical settings.

This review found that tree-based algorithms under supervised learning models, particularly random forest and gradient boosting performed, the best. Similarly, another review that evaluated prognosis in psychosis found that random forest was amongst the best performing algorithms [59]. Tree-based models can handle complex relationships between variables, large numbers of predictors, and missing data [60], and hence are appropriate for clinical studies. In contrast, Cho et al. [45] found that Support Vector Machines outperformed other models in diagnosing mental disorders. In terms of the predictive accuracy in diagnosing BD specifically, a recent review found that deep learning, specifically the convolutional neural network, performed the best [61]. The variations in the performance of machine learning models across studies are dependent on the unique strengths and characteristics of each model. For instance, the number and type of predictive variables involved can influence the performance of the different models as Support Vector Machine algorithms work well with sparse data while gradient boosting machine and random forest algorithms perform well with significant data [45]. Other reviews that evaluated the prognostic outcomes of mental disorders did not comment on the best AI algorithm used [19,62,63]. As Amanollahi et al. noted, appropriate comparisons of the accuracy of AI algorithms need to take into account the specific outcomes and predictors examined across studies [19].

With regard to functioning, consistent with the current literature, higher educational level [64] and fewer hospitalisations [64,65] were predictive of better cognitive functioning, while childhood abuse and neglect were predictive of poorer functioning. Other previously reported positive predictors included preserved cognitive ability [64] and lithium therapy within first ten years of diagnosis [65] while negative predictors included substance use and presence of comorbidities [65], such as diagnosis of depression, mixed states, and presence of personality disorder [64]. Whilst a study in this review found that age of onset, illness duration, and family history of mood disorders were not discriminatory of cognitive functioning, these variables were associated with illness severity in BD in other studies [66,67].

In terms of association with affective states, this review found that pause features, vocalisation, and postures in speech and video recordings were associated with different illness states. Consistent with earlier studies, physiological data from wearables such as activity and sleep levels were useful variables to distinguish illness states [17]. Further work needs be carried out to evaluate the comparative predictive accuracy of individual versus combinatorial data stream in differentiating changing affective states.

In terms of relapse, findings from machine learning (ML) models both align with and diverge from the broader clinical literature. Sociodemographic variables such as age and gender demonstrated effects across studies. Younger age was identified as a relapse predictor, and is consistent with epidemiological data, which found that older age is associated with more favourable long-term outcomes, likely due to greater illness insight and treatment stability in older adults [68]. The mixed associations with gender mirror broader reviews, indicating that gender effects are modest and context-dependent, frequently mediated by clinical and psychosocial factors rather than acting as direct predictors of relapse itself [68].

Clinical and symptomatic predictors were more concordant with existing knowledge. Prodromal and behavioural changes, including mood lability, sleep disturbance, psychomotor shifts, anxiety, and impaired thought processes, have long been recognised as early warning signs of mood episode recurrence (e.g., elevated unusual thought content preceding relapse) in BD [69]. Beyond traditional clinical markers, machine learning models incorporating neurobiological data (e.g., altered amygdala activity, changes in white matter volumes, disrupted functional connectivity) highlight the potential of integrating objective biomarkers into relapse risk stratification. These neuroimaging findings are consistent with illness models positing that affective dysregulation and reward circuitry abnormalities underpin mood instability in BD [70].

Importantly, digital phenotyping signals derived from video and wearable sensors, such as reduced head movement in depressive states, electrodermal changes, heart rate, sleep patterns, activity acceleration, and circadian rhythm alterations, represent emerging objective predictors that complement subjective symptom monitoring. Some evidence suggests that physiological markers from wearables may detect relapse risk states that precede clinical presentation, aligning with research advocating for physiological monitoring to enhance predictive accuracy beyond self-reported symptoms [71]. Altogether, these findings suggest that multimodal predictive models—that is, combining sociodemographic, clinical, neurobiological, and digital phenotyping features—may offer the most promise for relapse prediction in BD, while acknowledging the need for larger, diverse datasets and external validation to improve generalisability and clinical utility.

There are several limitations of this review. First, the search strategy was limited to studies published in English, which may have resulted in the exclusion of relevant studies published in other languages. Second, this review paper may be vulnerable to publication bias as grey literature was excluded. Third, despite efforts to minimise selection bias through systematic screening of articles, some degree of subjectivity in the study selection process cannot be entirely excluded. Finally, there was considerable heterogeneity among the included studies in terms of methodology (e.g., datasets and sample sizes), study populations (e.g., clinical background), and outcomes examined. The small sample size in certain studies also limits the statistical strength of the findings. The studies also utilised different predictive variables, machine learning algorithms, performance metrics, and validation approaches, preventing quantitative synthesis such as meta-analysis. Consequently, the findings of this review were synthesised narratively. Future research could look into collaborative studies involving larger samples from multiple sites to enhance generalisability of research findings. Studies may also want to examine multimodal predictive models, that is, combining sociodemographic, clinical, neurobiological, and digital phenotyping features for the prediction of clinical outcomes in BD.

In conclusion, existing studies demonstrate the growing potential of machine learning methods to predict key clinical trajectories in BD, including functional outcomes, affective states, and relapse risk, using diverse clinical, biological, and digital data sources. However, current evidence remains heterogeneous and largely exploratory. Well-designed longitudinal studies with larger, more representative samples and external validation are needed to refine and confirm clinically meaningful predictors. Such advances could enable earlier identification of individuals at heightened risk of poor prognosis, support more timely and targeted interventions, and inform personalised management strategies to improve long-term outcomes in BD.

## Data Availability

No new data were created or analysed in this study. Data sharing is not applicable to this article.

## Supplementary Materials

The following supporting information can be downloaded at: https://www.mdpi.com/article/10.3390/brainsci16040415/s1, Table S1: Search keywords; Table S2: Quality Appraisal.
