# Supplementary material for: Accuracy of Machine Learning Models in Predicting Clinical Outcomes in Bipolar Disorder: A Systematic Review
Source: Brain Sci. 2026 Apr 15;16(4):415. doi: 10.3390/brainsci16040415 (PMC13114975; doi:10.3390/brainsci16040415)
Supplement: Supplementary file 1 [file brainsci-16-00415-s001.zip › brainsci-4191109-supplementary.pdf]

Table S1: Search keywords

Databases	Keywords
**CINAHL**	AB ((artificial intelligence) OR (machine learning) OR (natural language processing) OR (neural network) or (data science) OR (deep learning) OR (digital biomarker*) OR (digital phenotyping)) AND AB (bipolar*) AND AB (((hospitali*) OR relapse OR recovery OR function* OR remiss* OR (illness state))
**Cochrane Library**	(artificial intelligence) OR (machine learning) OR (natural language processing) OR (neural network) or (data science) OR (deep learning) OR (digital biomarker*) OR (digital phenotyping) in Title Abstract Keyword AND (bipolar*) in Title Abstract Keyword AND (hospitali*) OR relapse OR recovery OR function* OR remiss* OR (illness state) in Title Abstract Keyword - (Word variations have been searched)
**Embase**	('artificial intelligence':ab,ti OR 'machine learning':ab,ti OR 'natural language processing':ab,ti OR 'neural network':ab,ti OR 'data science':ab,ti OR 'deep learning':ab,ti OR 'digital biomarker*':ab,ti OR 'digital phenotyping':ab,ti) AND bipolar*:ab,ti AND (hospitali*:ti,ab,kw OR relapse:ti,ab,kw OR recovery:ti,ab,kw OR function*:ti,ab,kw OR remiss*:ti,ab,kw OR 'illness state':ti,ab,kw)
**PubMed**	(((artificial intelligence[Title/Abstract]) OR (machine learning[Title/Abstract]) OR (natural language processing[Title/Abstract]) OR (neural network[Title/Abstract]) OR (data science[Title/Abstract]) OR (deep learning[Title/Abstract]) OR (digital biomarker*[Title/Abstract]) OR (digital phenotyping[Title/Abstract])) AND (bipolar*[Title/Abstract])) AND ((hospitali*[Title/Abstract]) OR relapse[Title/Abstract] OR recovery[Title/Abstract] OR function*[Title/Abstract] OR remiss*[Title/Abstract] OR (illness state[Title/Abstract]))
**PsycInfo**	((artificial intelligence) OR (machine learning) OR (natural language processing) OR (neural network) or (data science) OR (deep learning) OR (digital biomarker*) OR (digital phenotyping) and bipolar* and (hospitali*) OR relapse OR recovery OR function* OR remiss* OR (illness state)).ab.
**Science Direct**	(artificial intelligence) OR (machine learning) OR (natural language processing) OR (neural network) or (data science) OR (deep learning) OR (digital biomarker) OR (digital phenotyping) in title, abstract or author-specified keywords field and bipolar in title field.
**Scopus**	(ABS ((artificial AND intelligence) OR (machine AND learning) OR (natural AND language AND processing) OR (neural AND network) OR (data AND science) OR (deep AND learning) OR (digital AND biomarker*) OR (digital AND phenotyping)) AND ABS (bipolar*) AND (ABS ((hospitali*) OR relapse OR recovery OR function* OR remiss* OR (illness state))

Table S2: Quality Appraisal

Authors	Consecutive/ random sample	Case control design avoided	Avoid inappropriate exclusions	Index test results interpreted independently	Was threshold pre-specified?	Correct classification by reference standard?	Reference standard results interpreted independently	Appropriate interval between index and reference standard	Did all patients receive the same reference standard?	Were all patients included in analysis?	Total score
Abaei et al., 2020	+	-	+	NA	++	++	NA	NA	++	++	4
AbaeiKoupaei et al., 2020	+	-	+	NA	++	++	NA	NA	++	++	4
Amiriparian et al., 2019	+	-	+	NA	++	++	NA	NA	++	++	4
Anmella et al., 2023	+	-	++	NA	++	++	NA	NA	++	++	5
Belizario et al., 2019	+	+	++	NA	++	++	NA	NA	++	++	5
Bennett et al., 2022	++	-	++	NA	++	++	NA	NA	++	++	6
Borges-Junior et al., 2018	+	-	+	NA	++	++	NA	NA	++	++	4
Cho et al., 2019	+	++	+	NA	++	+	NA	NA	++	++	4
Cote-Allard et al., 2022	+	-	++	NA	++	++	NA	NA	++	++	5
Crocamo et al., 2024	+	+	+	NA	++	++	NA	NA	++	++	4
Ebrahim et al., 2018	+	-	+	NA	++	++	NA	NA	++	++	4
Edgcomb et al., 2019	+	+	++	NA	++	++	NA	NA	++	++	5
Gideon et al., 2016	+	+	+	NA	++	++	NA	NA	++	++	4
Grunerbl et al., 2014	+	++	+	NA	++	++	NA	NA	++	++	5

Authors	Consecutive/ random sample	Case control design avoided	Avoid inappropriate exclusions	Index test results interpreted independently	Was threshold pre-specified?	Correct classification by reference standard?	Reference standard results interpreted independently	Appropriate interval between index and reference standard	Did all patients receive the same reference standard?	Were all patients included in analysis?	Total score
Horigome et al., 2020	+	++	+	NA	++	++	NA	NA	++	++	5
Jakobsen et al., 2022	-	++	++	NA	++	++	NA	NA	++	++	6
Karam et al., 2014	+	+	+	NA	++	++	NA	NA	++	++	4
Kjærstad et al., 2022	+	-	+	NA	++	++	NA	NA	++	++	4
Lee et al., 2023	+	++	+	NA	++	++	NA	NA	++	++	5
Li et al., 2019	+	-	+	NA	++	++	NA	NA	++	++	4
Maxhuni et al., 2016	+	-	+	NA	++	++	NA	NA	++	++	4
Miola et al., 2024	++	++	+	NA	++	++	NA	NA	++	++	6
Muaremi et al., 2014	+	++	+	NA	++	++	NA	NA	++	++	5
Murugavel et al., 2025	+	+	+	NA	++	++	NA	NA	++	++	4
Pan et al., 2018	+	-	+	NA	++	++	NA	NA	++	++	4
Palacios-Ariza et al., 2023	+	++	++	NA	++	++	NA	NA	++	++	6
Palau et al., 2023	+	++	++	NA	++	++	NA	NA	++	++	6
Panagiotou et al., 2022	+	++	+	NA	++	++	NA	NA	++	++	5
Rabelo-da-Ponte et al., 2022	+	-	++	NA	++	++	NA	NA	++	++	5

Authors	Consecutive/ random sample	Case control design avoided	Avoid inappropriate exclusions	Index test results interpreted independently	Was threshold pre-specified?	Correct classification by reference standard?	Reference standard results interpreted independently	Appropriate interval between index and reference standard	Did all patients receive the same reference standard?	Were all patients included in analysis?	Total score
Rodrigues de Aguiar et al., 2023	++	++	++	NA	++	++	NA	NA	++	++	7
Salem et al., 2019	++	++	++	NA	++	++	NA	NA	++	++	7
Salvini et al., 2015	+	-	+	NA	++	++	NA	NA	++	++	4
Sartori et al., 2018	+	-	+	NA	++	++	NA	NA	++	++	4
Sankar et al., 2023	+	+	+	NA	++	++	NA	NA	++	++	4
Shao et al., 2023	+	-	+	NA	++	++	NA	NA	++	++	4
Valenza et al., 2013	+	+	+	NA	++	++	NA	NA	++	++	4
Xing et al., 2018	+	-	+	NA	++	++	NA	NA	++	++	4
Yang et al., 2018	+	-	+	NA	++	++	NA	NA	++	++	4
Zlatintsi et al., 2022	+	++	+	NA	++	++	NA	NA	++	++	5
Zhu et al., 2023	++	+	++	NA	++	++	NA	NA	++	++	6
